# Pathology of Delirium Tremens, Erysipelas, Cholera Morbus, &c. Founded on the Blood's Vitality

**Published:** 1825-08

**Authors:** John Jones

**Affiliations:** Surgeon, &c.


ART. II.—*Pathology of Delirium Tremens, Erysipelas, Cholera Morbus, &c. founded on the Blood's Vitality.* By JOHN JONES, Esq. Surgeon, &c.

[Concluded from page 12.]

THE third division of diseases connected with the blood's vitality are characterised by morbid irritability, or increased action accompanied with debility. In a former paper, morbid irritability was defined to be a peculiar susceptibility for impressions, by which pain is readily induced, or the system unusually agitated, by the application of stimuli. It is either connected with original formation, or is the effect of disease; and affects the whole system, or only particular tissues. It particularly predominates in the system in the sanguine temperament,—generally accompanies protracted disease, either chronic or acute, especially the latter, as the phlegmasiæ, &c.; and the febrile state by which they are attended is frequently continued by its influence, long after the local affection, on which the fever originally depended, has subsided. Particular tissues are either naturally under its influence, or become so in consequence of previous disease. Thus, some individuals are born with irritable bowels, in whom much less medicine is found to operate than is usually required by others; and, when inflammation has once occurred, a susceptibility in the part to be similarly affected continues for long afterwards. This is remarkably the case with the mucous membrane; and we accordingly find catarrh, pneumonia, enteritis, ophthalmia, &c. are induced, in after attacks, by much slighter exciting causes than were required for their first production.

For the well-being of the animal machine, it is necessary not only that vitality should be communicated from the blood, through the medium of the nerves, but that the various tissues should be also endowed with a *capacity* duly to appropriate the vitality they receive. Thus, if the muscular fibre is weak, although it might be abundantly supplied with nervous energy, the animal powers will be also weak: for, like a watch, however ingenious the mechanism, if the materials of which it is composed are not good, the machine is imperfect in its operations. The collective powers of life, therefore, reciprocally depend on each other, and any causes which tend to destroy this mutual dependence must lead to disease. Arterial action, in whatever way excited, is attended with a corresponding expenditure of nervous energy, which renders the nerves morbidly sensible. If the collective powers of life are *vigorous*, what has been called the phlogistic diathesis prevails, and the diseases with which it is connected are attended by a reproduction of vitality, in some measure proportionate to its morbid expenditure. If *weak*, diseases characterised by morbid irritability are the consequence, in which increased arterial action is attended by an expenditure of nervous power greater than can be supported by the system, and exhaustion is soon produced, if the causes of disease be not removed. If venous congestion of any of the internal organs should at the same time exist, the debility is proportionably increased, and exhaustion accelerated.

The following diseases are illustrative of the class connected with morbid irritability:—1. Delirium tremens; 2, Erysipelas; 3, Cholera morbus.

1. *Delirium tremens.*—Till the year 1813, this disease was confounded with phrenitis, and we are indebted to Dr. Sutton for a correct description of its symptoms as a distinct disease, and likewise for recommending opium as its appropriate remedy. It most frequently occurs in those who are addicted to the intemperate use of spirits. When a predisposition is thus induced, it is readily excited, and the immediate cause is, perhaps, an unusual excess in debauch. This is followed by prostration of strength and staggering in the gait; the intellect soon becomes confused; the patient talks incoherently to himself; his delirious wanderings are fixed upon his own imaginary greatness,—he is a king, or some great man surrounded by his slaves and dependants: he can, however, be roused from his reveries, and answers questions correctly, but soon relapses into the same state. As the disease advances, the hallucinations become more constant, and attended with watchfulness. The hands are remarkably tremulous; skin seldom exceeds the natural temperature, and is bedewed with a clammy moisture; pulse weak, and not much accelerated at first, but afterwards becomes quick and tremulous; tongue moist, furred and tremulous when protruded.

In delirium tremens, the existence of positive inflammation is not indicated; but there is such a determination of arterial blood to the brain, and consequent expenditure of nervous energy, as materially to derange the cerebral functions, and those of the nervous system in general. Such a cause readily becomes effective, if the energies of the constitution have been previously weakened by habitual intemperance, and the disease becomes modified accordingly. If at the same time the abdominal viscera are in a state of venous congestion, which there is reason to believe is frequently the case, the general debility is increased, and the affection of the brain aggravated by, if not produced sympathetically with, such organic derangement.

*Treatment.*—Large and repeated doses of opium, given till the agitation of the system is subdued and sleep procured, are the principal means recommended by Dr. Sutton. When the hepatic and chylopoietic functions are impaired, alterative doses

Leave off drinking of blue-pill, conjoined with gentle aperients, are also necessary; and, to lessen sanguineous determinations to the head, leeches to the temples, and blisters on the nape of the neck or behind the ears, together with cold applications to the scalp.

As diminished tone of the system forms a principal character of this disease, active depletion is wholly inadmissible; and evacuants are therefore to be used with great caution, lest we add still further to the depression, and accelerate a fatal exhaustion.

*Erysipelas.*—The inflammation which characterises this disease peculiarly depends on morbid irritability. Those who are most subject to it are children, the aged, or such as have impaired their constitution by habits of intemperance, or are naturally delicate. In individuals of this description, slight local causes will produce it. Sometimes it occurs sympathetically with organic derangement, and seems, like gout, an effort of the constitution to relieve itself. Not unfrequently it supersedes a fit of the gout, and is thus strongly corroborative of both diseases depending on the same cause.

Whatever might be the exciting cause of erysipelas, and whether symptomatic or idiopathic, it can only be considered a modification of inflammatory action, and the modifying cause—morbid irritability, either of the whole system or only of the cuticular tissue. This is an important distinction, and in some measure reconciles the discordant views which exist, both of the pathology and treatment of this disease. When it is connected with irritability of the system, the vital energies are generally either naturally weak, or impaired by age, intemperate living, or previous disease. The accompanying fever is of the typhoid type, and the inflammation has a tendency to terminate in mortification. When the modifying cause exists only in the skin, the subjects of the disease are, for the most part, the young and plethoric; the symptoms of febrile excitement are strongly marked; and the inflammation often partakes both of an erysipelatous and phlegmonous character, terminating in suppuration.

When the irritability of the skin is great, local injuries are frequently attended with erysipelatous inflammation, which occasionally assumes the erratic character in a remarkable degree, which so peculiarly distinguishes this disease. An elderly woman, of a robust constitution, and accustomed to hard labour, was attacked with inflammation of one of her fingers: the pain was intense; the back of the hand soon became puffed, and the redness and swelling extended nearly to the axilla. I suspected that kind of whitlow which is seated in the theca of the flexor tendons, and immediately made an incision to the bone, which was followed by a little blood mixed with pus. Suppuration also occurred on the back of the hand, and the matter was discharged by an incision. In a few days, an abundant purulent discharge occurred from the first opening in the finger, as if it had communicated with another abscess. The free vent which had thus been given to the matter, although it relieved the urgent symptoms, did not remove the erysipelatous inflammation, which continued its progress from the arm to the shoulder, and successively attacked the face, breast, abdomen, back, and finally expended itself in the thighs.

*Treatment.*—From the above view of the pathology of erysipelas, it is obvious that the treatment must be regulated by the following considerations:—1. Whether it is connected with morbid irritability of the system, or only of the skin. 2. Whether it is excited by causes operating on the skin alone, or is symptomatic of functional derangement of the internal organs.

If the energies of the system are impaired, and there is reason to suspect that the inflammatory affection is sympathetically produced, local applications should be used with caution, lest, by repelling the inflammation, we interfere with a salutary process of nature, which she has chosen for relieving the constitution; although, as observed by Cullen, when situated on the extremities, it is rarely translated to internal parts. As the functions of the abdominal viscera are deranged in consequence of venous congestion, of which the local affection is only a symptom, the continued employment of aperients, together with alterative doses of blue-pill, is necessary; and, at the same time, the accompanying pain should be allayed by opiates frequently repeated. If symptoms of inflammation should occur in any of the internal organs, which is sometimes the case, it is only to be subdued by the cautious employment of bleeding, either locally or from the system, together with the application of blisters, &c. When the disease affects the young and plethoric, and the pyrexial symptoms are strongly marked, the benefit derived from depletion is generally in proportion to the freedom with which it is employed. Some interesting cases are published in the Edinburgh Medical and Surgical Journal for October 1821, communicated by Dr. A. Duncan, jun. in which venesection and purgatives, carried to an extent which had been previously considered inadmissible, were remarkably successful.

When the disease is idiopathic, or arises from acrid applications to an irritable skin, it is generally a trifling complaint, and is readily subdued by emollients or cooling lotions; although, if the vital energies are impaired, mortification is apt to be induced, which should be guarded against by the free exhibition of bark and generous diet.

*Cholera Morbus.*—This disease is characterised by extraordinary irritability of the mucous membrane of the stomach and intestines, produced by the combined agency of exciting and debilitating causes. It is greatly under the influence of the atmosphere, and occurs generally in hot and sultry weather; it is, therefore, most prevalent in tropical climates, where its fatality almost equals that of the plague: it is there so rapid in its progress, as hardly to admit time for the operation of remedies. It is, however, by no means unfrequent in our own climate, and I have known it terminate fatally in sixteen hours. In post-mortem examinations, the large internal veins, particularly of the mesentery, are generally found distended with black blood.

The heat of the weather can only be considered as predisposing to the disease; and, when such predisposition exists, it is readily induced by exposure to cold, or any other sedative agent operating on the surface. The blood is thus prevented from freely circulating in the cuticular vessels, and accumulates in the internal viscera, producing venous congestions, and the consequent nervous depression is usually so great as to prevent the occurrence of febrile reaction. This cause of debility is greatly increased by the secretions becoming vitiated, and producing that irritable state of the bowels so characteristic of the disease, and which exists to such an extent as to cause, perhaps, fifty or sixty evacuations in the course of a few hours. The stomach, partaking of the morbid state of the intestines, ejects most that is received into it; and at the same time there is severe tormina. Violent spasms occur in the muscles of the extremities, particularly the calves of the legs. The temperature of the surface is usually below the natural standard, and the extremities soon become cold. Pulse, from the commencement, is generally weak, and gradually becomes more feeble and intermittent. As the vital energies are depressed by the combined agency of venous congestion and morbid irritability, exhaustion is soon produced, and the disease terminates fatally, particularly in hot climates (as already observed), with awful rapidity.

Cullen describes cholera as depending wholly on a redundancy of bile, which produces the violent commotion of the bowels, from its vitiated quality. Later practitioners, however, who have had abundant opportunities in hot climates of observing the disease in its worst forms, consider the bile to be partially or wholly obstructed, and that the intestines are excited into such violent action by the vitiated state of their own secretions. Such different view of the pathology of cholera must, of course, lead to very opposite treatment.

*Treatment.*—The indications are—1, to allay the morbid irritability of the stomach and intestines; 2, to remove existing congestions, and thus to restore the balance of the circulation; 3, to support the energies of the system. The remedies usually employed for fulfilling the above indications, are opiates conjoined with calomel,—diluents and aperients,—fomentations and blisters. Venesection has also been used to a great extent by some practitioners in India, whose authority is entitled to respect; although, from the extreme depression accompanying the disease, it would appear, *a priori*, more calculated to extinguish the remaining energies, than to contribute to the recovery of the patient.

For allaying the inordinate action of the stomach and intestines, and relieving the violent spasms, opiates are indispensable, and should be given in large and repeated doses. In the Indian cholera, calomel has been extensively employed, and is found more entitled to confidence than any other remedy: when conjoined with opium, its effect of emulging the hepatic vessels is more certainly insured. Although active inflammation can hardly be supposed to exist in a disease so much characterised by debility, yet, as morbid irritability is the effect of arterial action, cautious depletory measures for removing such a state are admissible; and we find the application of leeches, and afterwards a large blister, to the abdomen, often beneficial. The vital energies are sometimes so much depressed, that nothing but the repeated exhibition of ardent spirits will prevent them from becoming wholly extinguished.

I have now arrived at the conclusion of the subject of this and the preceding papers, and, although the pathological opinions they contain are in many respects novel, I trust they have been sufficiently supported by facts and illustrations, as to entitle them to some consideration. I am aware that, to oppose established doctrines, might be justly deemed presumptuous; and, unless supported by the strongest evidence, the author of such innovations subjects himself to contempt. New doctrines, especially in medicine, have always to contend with prejudices founded upon preconceived notions, which have been imbibed by education and fostered by practice. Prejudice, however, is to be deprecated as incompatible with a genuine love of science: it renders us incapable of judging, with candour and impartiality, opinions at variance with our own, and of discovering any thing but imperfections in them, in opposition to the clearest evidence to the contrary. Although prejudice is opposed to the progress of science, yet it must be granted that a too-ready acquiescence in novel doctrines is equally adverse to medical improvement. Hypothetical reasonings, unless well supported by facts, are always to be received with great caution; for it is well known that they are often fallacious, even when possessing the greatest plausibility, and originating with men of unquestionable ability. We should, however, recollect that the late decided improvements in the practice of medicine, particularly in the treatment of fever, are owing, in a great measure, to individuals who discarded the hypothetical doctrines they had been taught,—divested themselves of the shackles of preconceived notions,—and were guided by matter-of-fact alone. The happy result proves that this independent mode of acting and thinking is entitled to our warmest approbation; although, at the same time, we should remember that it is the abuse, and not the use, of hypothesis which is to be deprecated. Whilst we blindly submit to the *ipse dixit* of those whom we have been accustomed to look upon as the oracles of physic, we neglect to exercise our own judgment. When hypothesis is confined to facts alone, and is not dictated by the flights of a wild imagination, it increases the practitioner's confidence in the propriety of his measures,—affords him the pleasing consciousness of acting upon rational principles,—and peculiarly distinguishes him from the presumptuous and illiterate charlatan.

